# Rituximab treatment for chronic steroid-dependent Henoch-Schonlein purpura: 8 cases and a review of the literature

**DOI:** 10.1186/s12969-018-0285-2

**Published:** 2018-11-14

**Authors:** Courtney B. Crayne, Esraa Eloseily, Melissa L. Mannion, Saji P. Azerf, Peter Weiser, Timothy Beukelman, Matthew L. Stoll, Daniel I. Feig, T. Prescott Atkinson, Randy Quentin Cron

**Affiliations:** 1 Department of Pediatrics, Division of Rheumatology, University of Alabama at Birmingham, 1600 7th Ave S, CPPN G10, Birmingham, AL 35233 USA; 2 School of Medicine, University of Alabama at Birmingham, 510 20th St S, #12, Birmingham, AL 35233 USA; 3 Division of Nephrology, Department of Pediatrics, University of Alabama at Birmingham, 1600 7th Ave S, ACC 516, Birmingham, AL 35233 USA; 4 Division of Allergy & Immunology, Department of Pediatrics, University of Alabama at Birmingham, 1600 7th Ave S, CPPN M20, Birmingham, AL 35233 USA

**Keywords:** Henoch-schonlein purpura, Rituximab, B-lymphocytes, IgA nephropathy

## Abstract

**Background:**

Henoch-Schonlein purpura (HSP) is a small vessel vasculitis that is characterized by non-thrombocytopenic purpura, abdominal pain, arthritis, and glomerulonephritis. Typically, HSP is self-limited requiring only supportive care, but more severe cases may require corticosteroid (CS) treatment. Rarely, a subset of these patients has persistent rash, arthritis, abdominal involvement, or renal disease despite treatment with CS, or has disease recurrence on CS tapering. Refractory HSP has been effectively treated with a variety of CS sparing therapies. For life-threatening refractory HSP, the B cell depleting agent, rituximab (RTX), has been reported as beneficial for children with substantial renal or central nervous system involvement. However, RTX use for children with less severe HSP, but chronic CS dependent disease refractory to CS sparing immunomodulatory agents, has been less well explored. Herein, we describe 8 children treated with RTX for chronic refractory HSP and report a reduction in recurrent hospitalizations and eventual CS discontinuation.

**Methods:**

This is a retrospective analysis of eight children who were treated with RTX for chronic CS dependent HSP during the years 2006–2014 at a single institution. A chart review of the electronic medical record was performed to determine the presenting symptoms, the type and duration of treatment received, and the number of hospitalizations prior to and after RTX. The number of hospitalizations and oral corticosteroid burden were analyzed using the Wilcoxon signed rank test.

**Results:**

Prior to receiving RTX, seven patients had at least one hospitalization for HSP (median 1.5, range 0–3). Following RTX, only two patients were hospitalized, each a single time for recurrent abdominal pain. The median oral CS burden was 0.345 mg/kg/day before RTX and 0 mg/kg/day at 6 months (*p* = 0.078), 1 year (*p* = 0.0625), and 2 years (*p* = 0.03) following RTX infusion. Seven out of eight children met remission criteria, defined as no active rash, arthritis, nephritis (hematuria and proteinuria), or gastrointestinal distress following RTX. No serious adverse events were noted.

**Conclusion:**

Overall, RTX effectively reduced the number of hospital admissions and oral CS burden. RTX also helped most all children achieve clinical remission. RTX appears to be an effective and safe alternative for chronic CS dependent and immunomodulatory refractory childhood HSP.

## Introduction

Henoch-Schonlein purpura (HSP) is a small vessel vasculitis that is characterized by non-thrombocytopenic purpura, abdominal pain, arthritis, and glomerulonephritis [1]. It affects approximately 1 in 5000 children annually and accounts for about half of all childhood vasculitides in the United States [2, 3]. Typically, HSP is self-limited requiring only supportive care, but more severe cases may require corticosteroid (CS) treatment [4]. Rarely, a subset of these patients has persistent rash, arthritis, abdominal involvement, or renal disease despite treatment with CS, or has disease recurrence on CS tapering. Frequency of disease relapse is not well-documented in children, but may be as high 30–40%, often occurring within 1–2 months following diagnosis [5]. CS remains the standard treatment for symptomatic HSP. A small subset of patients are unable to wean off CS and subsequently require long term CS or additional steroid-sparing agents for disease control [6].

While there is no consensus definition for refractory HSP, for the purposes of this study, refractory HSP was defined as recurrence of symptoms following taper of CS. Refractory HSP has been effectively treated with a variety of CS sparing immunomodulatory disease modifying anti-rheumatic drug (DMARD) therapies [7], including intravenous immunoglobulin (IVIg) [8], colchicine [9], cyclosporine (CSA) [10], methotrexate (MTX) [11], cyclophosphamide (CTX) [12], mycophenolate mofetil (MMF) [13], and azathioprine (AZA) [14]. Novel CS sparing and safe immune modulators are needed for those with chronic HSP and immunomodulatory refractory disease.

For severe life-threatening refractory HSP, the B cell depleting agent, rituximab (RTX), has been reported as beneficial for three children with substantial renal or central nervous system involvement [15, 16]. RTX is a monoclonal antibody that binds to CD20, which is expressed on human B cells and is expressed at a low level on a small subset of T cells [17–21]. After binding to CD20, it induces B cell depletion by complement- and antibody-mediated cytotoxicity, although some evidence suggests that some non-circulating tissue B cells bind RTX and are not depleted [22, 23]. Except for the occasional occurrence of mild-to-moderate infusion reactions that may be dependent on complement fixation via the Fc portion of the antibody, RTX is usually well tolerated [24–27]. However, serum sickness, agranulocytosis, fatal infections, including progressive multifocal leukoencephalopathy (PML), and death from other causes have been rarely associated [28–31]. In addition to its use in B cell malignancies, RTX is used in a variety of antibody-mediated autoimmune diseases, including systemic lupus erythematosus, rheumatoid arthritis, anti-neutrophil-cytoplasmic antibody-associated vasculitis, and others [32]. RTX appears to be an alternative intervention for patients with severe, life-threatening refractory HSP, and has been successfully used in adults with refractory HSP [16]. Herein, we describe eight children treated with RTX for less severe, but chronic refractory HSP and report a reduction in recurrent hospitalizations and eventual oral CS discontinuation.

## Methods

A retrospective chart review of children diagnosed with HSP treated with RTX was performed. Clinical, laboratory, pathology, treatment, side-effect, and outcome data were abstracted from the electronic medical records for all patients seen at Children’s of Alabama (CoA) with the diagnosis of HSP and who had received RTX therapy in the years 2006 through 2014 and had at least two years of follow-up following initial RTX infusion. Previously reported patients were excluded [15]. One patient was excluded for noncompliance and inconsistent follow-up. Laboratory data (e.g., inflammatory markers, complete blood counts) were inconsistently obtained and therefore not included in this study.

For the purposes of this study, chronic CS use was defined as ≥6 weeks of daily oral CS intake, and refractory HSP was defined as CS dependent and/or DMARD refractory disease. There were no restrictions on DMARD or dose. Remission was defined as no active rash, arthritis, nephritis (hematuria or proteinuria) [5], or gastrointestinal distress. RTX was given at the treating physician’s discretion for prolonged CS use, DMARD-refractory, or recurrent hospitalizations for HSP. Each round of intravenous RTX was given as 2 doses of 750 mg/m^2^ (maximum of one gram) per dose, given 2 weeks apart [33]. RTX was given in conjunction with high dose intravenous pulse CS (30 mg/kg, maximum of one gram) to decrease the risk of an allergic reaction to RTX [34]. The number of hospitalizations for HSP before and after the administration of the first course of RTX were compared using the Wilcoxon signed rank test. Oral CS burden (mg/kg/day) at time zero, 6 months, 1 year, and 2 years following initial RTX were compared using the Wilcoxon signed rank test. Significance threshold was set at *p* < 0.05. Institutional Review Board approval was obtained from the University of Alabama at Birmingham.

## Results

Eight patients diagnosed with HSP and treated with RTX met the inclusion criteria for this study. There were five males and three females (ages 2 months to 16 years at disease onset), seven of whom were Caucasian and one of whom was Hispanic (Table 1). All had non-thrombocytopenic purpura and renal (hematuria and/or proteinuria) involvement, seven had gastrointestinal involvement (severe cramping abdominal pain, hematochezia, or associated iron deficiency anemia), and two had arthritis, defined as swelling within a joint or limitation in the range of joint movement with joint pain or tenderness [35]. Six of the eight had renal biopsies confirming IgA nephropathy, ranging from WHO Class I to III/IV (Table 1). None of these eight HSP patients had severe, life-threatening or central nervous system disease, although all of them had varying degrees of non-progressive renal involvement, including one on dialysis. In addition, prior to RTX, seven of the eight children had significant gastrointestinal distress, frequently triggering re-hospitalization (Table 1).

**Table 1:** Demographics of Sample Cohort

Patient #	1	2	3	4	5	6	7	8
Age at diagnosis	2 months	16 years	8 years	5 years	14 years	8 years	5 years	13 years
Sex	Male	Male	Male	Female	Male	Male	Female	Female
Ethnicity	Caucasian	Caucasian	Caucasian	Caucasian	Caucasian	Caucasian	Hispanic	Caucasian
Palpable purpura	Yes	Yes	Yes	Yes	Yes	Yes	Yes	Yes
Hematuria and/or proteinuria	Yes	Yes	Yes	Yes	Yes	Yes	Yes	Yes
Renal biopsy pathology	Not done	WHO Class I	WHO Class III	WHO Class III	WHO Class III/IV	WHO Class II	WHO Class II	Not done
Arthritis	No	No	Yes	No	No	No	No	Yes
GI bleed/distress	No	Yes	Yes	Yes	Yes	Yes	Yes	Yes
Long term CS use	Yes	Yes	No	Yes	Yes	Yes	Yes	Yes
Immuno-modulators (Months of DMARD prior to 1st RTX)	MTX [2]; MMF [4]	MTX [1]	AZA [4]	CTX (1 dose)	None	MTX [2]; MMF [7]	MTX [2]; MMF (post); IVIG (post)	MTX (> 3 years intermittent)

*AZA* azathioprine, *CS* corticosteroid, *CTX* cyclophosphamide, *GI* gastrointestinal, *IVIG* intravenous immunoglobulin, *MMF* mycophenolate mofetil, *MTX* methotrexate, *DMARD* disease modifying anti-rheumatic drug

Six of the children required long term daily oral CS therapy for ≥6 weeks. Six failed to respond to immunomodulatory therapy in the absence of daily oral CS treatment, and one failed DMARD therapy but was not on daily CS therapy. Patient 5 received RTX prior to trial of DMARDs due to end-stage renal disease requiring dialysis while on daily CS therapy. Patients received conventional DMARDs for at least one month prior to RTX (Table 1). The median oral CS burden prior to first RTX infusion was 0.345 mg/kg/day (range 0–1.28). Median oral CS burden was zero mg/kg/day at 6 months (range 0–0.5; *p* = 0.078), 1 year (range 0–0.2; *p* = 0.0625), and 2 years (range 0; *p* = 0.03) following initial RTX infusion (Table 2).

**Table 2:** Oral CS burden (mg/kg/day) change

Time from 1st RTX	Median	Range	Significance from *t* = 0
t = 0	0.345	0–1.28	–
*t* = 6 months	0	0–0.5	*p* = 0.078
*t* = 1 year	0	0–0.2	*p* = 0.0625
*t* = 2 years	0	0	*p* = 0.03

*RTX* rituximab; *p*-value calculated using paired Wilcoxon signed rank test; significance level p < 0.05

Four of the HSP patients underwent a single round (2 doses, 2 weeks apart) of RTX therapy, one child received 2 rounds, two children received 3 rounds, and one child underwent 6 rounds (Table 2). B-cell depletion data was available for seven of the cohort, and CD19 counts were notably reduced after the first rounds of RTX (undetectable in 4).

Median time to first RTX infusion was 16 months (range 1.5–96), and median number of RTX rounds (2 doses, 2 weeks apart) was 1.5 (range 1–6) (Table 3). Four of the eight patients received only one course of RTX with four children requiring more than 2 rounds to wean off CS. Prior to receiving RTX, seven patients had at least one hospitalization for HSP (median 1.5, range 0–3). Following RTX, only two patients were hospitalized, each a single time for recurrent abdominal pain (Table 3).

**Table 3:** Rituximab response distribution

Patient #	1	2	3	4	5	6	7	8	Median
Months to 1st RTX Infusion	26	1.5	27	3	7	25	3	96	16
Number of rounds of RTX (2 doses/round)	3	2	1	1	1	1	6	3	1.5
Hospitalizations for HSP prior to RTX	2	1	1	2	1	3	2	0	1.5
Hospitalizations for HSP post RTX	0	1	1	0	0	0	0	0	0
Months in remission as of July 2016	70	73	49	60	66	91	1	N/A	63

*HSP* Henoch-Schonlein purpura, *RTX* rituximab

In six of the eight patients, RTX effectively eliminated B cells as detected by CD19 count and resulted in rapid clinical improvement with the resolution of skin, joint, and gastrointestinal symptoms. One patient required scheduled IVIg infusion for replacement therapy and daily MMF for maintenance but was clinically in remission. One patient continued to receive RTX infusions in combination with MMF for ongoing abdominal complaints and therefore did not meet criteria for remission. Therefore, seven of the eight patients were in remission following RTX completion (range 1 to 91 months, median 63 months). Additionally, the one child who did not achieve remission at time of study completion (July 2016) was successfully weaned off CS and thus spared the burden of chronic CS usage.

High dose intravenous CS were given in conjunction with each RTX infusion, and there were no serious adverse events for these children after RTX therapy. More detailed safety data on this cohort is presented elsewhere as part of a larger series of RTX treated patients with a variety of rheumatic diagnoses [36]. Ultimately, RTX was well tolerated and allowed for sparing of CS with all patients off CS at time of this writing.

## Discussion

Although HSP tends to be a self-limited disease, it has been suggested the recurrence rate is about 16% [6]. Treatment for CS dependent and DMARD refractory HSP, however, can be challenging. There are reasons to think that B cell depletion may be an attractive for treating CS dependent DMARD refractory HSP. These include the role of B cells serving as antigen presenting cells to T cells, both in terms of T cell priming and co-stimulation. Moreover, over time decreased levels of circulating IgA may help diminish disease pathology in HSP [37]. In 2009, Donnithorne et al. described the use of RTX in 3 cases of severe refractory chronic HSP. All three cases had gastrointestinal involvement, 2 had IgA nephropathy, and two had CNS involvement, one with vasculitis (Table 4). Two of the three cases were treated with CTX without response. All three cases were unable to taper CS initially. All three eventually achieved remission with RTX treatment and were able to taper CS [15].

**Table 4:** Previously published cases treated with RTX for severe refractory HSP

Authors	Year	HSP patients
Donnithorne et el. [15]	2009	3 children ages 17 (male), 14 (male), 10 (female) years old with severe chronic refractory HSP
Pillebout et al. [38]	2011	22-year-old male with severe recurrent skin manifestations and nephritis in biopsy (RTX as only immunomodulation)
Kostik et al. [39]	2013	7-year-old female with severe relapsing HSP with initial response followed by failure 2.5 years later
Tosounindou et al. [40]	2013	61-year-old female with intractable GI manifestations that required repeated courses of RTX to maintain remission
El-Husseini et al. [41]	2013	27-year-old male with drug-induced HSPN
Ishiguro et al. [42]	2013	68-year-old female with refractory purpura nephritis
Pindi Sala [43]	2014	49-year-old female with CS dependent HSP
Bellan et al. [16]	2016	57-year-old female with recurrent purpura and proteinuria refractory to CTX and CS

*CS* corticosteroid, *GI* gastrointestinal, *HSP* Henoch-Schonlein purpura, *RTX* rituximab

Since that first report, others have reported on the use of RTX treatment for chronic HSP (Table 4). In 2011, Pillebout et al. reported a successful outcome using RTX as the only immunomodulation in a 22-year-old patient who had severe recurrent skin manifestations and HSP nephritis (HSPN) on renal biopsy [38]. In 2013, Kostik et al. reported a case of 7-year-old girl with a severe relapsing HSP who presented initially with skin and gastrointestinal symptoms and responded to CS, heparin, and plasma exchange, but later had HSPN WHO type IV, refractory to CS, CTX, MMF, low molecular weight heparin, and dypiridamol. The patient then dramatically responded to RTX and was able to taper CS, requiring CTX and AZA for maintenance. Unfortunately, 2.5 years later, the patient had deteriorating kidney function and subsequent treatment with RTX was not effective [39]. Also in 2013, Tosounindou et al. reported a 61 year old female with HSP who had intractable gastrointestinal (GI) involvement treated with repeat courses of RTX used to maintain remission over 9 years [40]. RTX use for refractory HSPN was also reported in 2013 by El-Husseini et al. in a 27-year-old man who had drug induced HSPN and had previously failed to respond to CS, CTX, and plasmapheresis. His renal and extra-renal symptoms improved after RTX treatment [41]. Similarly, Ishiguro et al. reported a 68-year-old woman who had purpura nephritis associated with nephrotic syndrome who failed standard treatment with CS and intravenous CTX with complete remission following RTX treatment [42]. In 2014, Pindi Sala presented a case of 49-year-old woman who developed HSP 8 years after kidney transplant for segmental glomerular hyalinosis, while being on tacrolimus and AZA. The vasculitis involved skin and the sciatic peroneal nerve branch. She was CS dependent as she had four relapses. Successful outcome was observed during two years of follow-up after RTX treatment [43]. In 2016, Bellan et al. described a 57-year-old woman who had recurrent HSP flares and moderate proteinuria since first diagnosis 20 years prior. She had renal pathology confirming IgA nephropathy and persistent purpuric rash with proteinuria despite treatment with cyclophosphamide. She had complete remission off all medications following RTX treatments, and at time of study publication remained in remission 8 years following last RTX [16]. While RTX use for severe refractory HSP is well-reported (Table 4), its use in children with non-life threatening HSP, but chronic CS dependent disease refractory to CS sparing immunomodulatory DMARD agents, has been less well explored.

We now report a cohort of eight children with non-life threatening, but refractory, chronic CS dependent HSP who benefited from RTX therapy without any noted adverse effects or complications. While just outside the 0.05 cutoff of statistical significance, a decrease in the number of hospitalizations following initial RTX infusion was evident. Further, oral CS burden trended toward statistical significance by 2 years with all patients able to discontinue use of daily CS. Additionally, RTX allowed for the elimination of other immunosuppressive therapy in six of the eight children and helped seven of them achieve and maintain remission for 1 to 91 months. The mechanism by which RTX resulted in disease improvement was not evaluated in this study. However, as the pathology involves IgA deposition, the loss of IgA-producing B cells likely contributes. Additionally, B cells perform additional functions in autoimmune diseases, such as cytokine production and antigen presentation [37].

This study is limited by its retrospective nature and small sample size, as well as the lack of a standardized approach to use of RTX therapy at our institution. Nevertheless, this cohort combined with previously reported data suggest RTX is a promising treatment for CS dependent chronic HSP, in patients with both severe and less severe refractory disease. This appears particularly valuable for decreasing CS burden preventing repeated hospitalizations. The response to RTX in these eight patients identifies the need to conduct prospective studies to further evaluate its benefit in treating chronic CS dependent HSP.

## Conclusions

Overall, RTX effectively reduced the number of hospital admissions and oral CS burden in children with less severe refractory HSP. RTX also helped most all children achieve clinical remission. RTX appears to be an effective and safe alternative for less severe but chronic CS dependent and immunomodulatory refractory childhood HSP. Future studies are needed to determine treatment initiation and duration recommendations.

## Abbreviations

AZA: Azathioprine
CS: Corticosteroid
CSA: Cyclosporine
CTX: Cyclophosphamide
DMARD: Disease modifying anti-rheumatic drug
HSP: Henoch-Schonlein purpura
HSPN: HSP nephritis
IVIg: Intravenous immunoglobulin
MMF: Mycophenolate mofetil
MTX: Methotrexate
RTX: Rituximab

## References

[1] Saulsbury FT (2007). Clinical update: Henoch-Schonlein purpura. Lancet.

[2] Bowyer S, Roettcher P (1996). Pediatric rheumatology clinic populations in the United States: results of a 3 year survey. Pediatric rheumatology database research group. J Rheumatol.

[3] Rostoker G (2001). Schonlein-henoch purpura in children and adults: diagnosis, pathophysiology and management. BioDrugs.

[4] Weiss PF, Klink AJ, Localio R, Hall M, Hexem K, Burnham JM (2010). Corticosteroids may improve clinical outcomes during hospitalization for Henoch-Schönlein purpura. Pediatrics.

[5] Calvo-Rio V, Hernandez JL, Ortiz-Sanjuan F, Loricera J, Palmou-Fontana N, Gonzalez-Vela MC (2016). Relapses in patients with Henoch-Schonlein purpura: analysis of 417 patients from a single center. Medicine (Baltimore).

[6] Lei WT, Tsai PL, Chu SH, Kao YH, Lin CY, Fang LC (2018). Incidence and risk factors for recurrent Henoch-Schonlein purpura in children from a 16-year nationwide database. Pediatr Rheumatol Online J.

[7] Chartapisak W, Opastiraku S, Willis NS, Craig JC, Hodson EM (2009). Prevention and treatment of renal disease in Henoch-Schonlein purpura: a systematic review. Arch Dis Child.

[8] Lamireau T, Rebouissoux L, Hehunstre JP (2001). Intravenous immunoglobulin therapy for severe digestive manifestations of Henoch-Schonlein purpura. Acta Paediatr.

[9] Saulsbury FT (2009). Successful treatment of prolonged Henoch-Schonlein purpura with colchicine. Clin Pediatr.

[10] Jauhola O, Ronkainen J, Autio-Harmainen H, Koskimies O, Ala-Houhala M, Arikoski P (2011). Cyclosporine a vs. methylprednisolone for Henoch-Schonlein nephritis: a randomized trial. Pediatr Nephrol.

[11] Rettig P, Cron RQ (2003). Methotrexate used as a steroid-sparing agent in non-renal chronic Henoch-Schonlein purpura. Clin Exp Rheumatol.

[12] Edstrom Halling S, Soderberg MP, Berg UB (2009). Treatment of severe Henoch-Schonlein and immunoglobulin a nephritis. A single center experience. Pediatr Nephrol.

[13] Nikibakhsh AA, Mahmoodzadeh H, Karamyyar M, Hejazi S, Noroozi M, Macooie AA (2010). Treatment of complicated henoch-schonlein purpura with mycophenolate mofetil: a retrospective case series report. Int J Rheumatol.

[14] Foster BJ, Bernard C, Drummond KN, Sharma AK (2000). Effective therapy for severe Henoch-Schonlein purpura nephritis with prednisone and azathioprine: a clinical and histopathologic study. J Pediatr.

[15] Donnithorne KJ, Atkinson TP, Hinze CH, Nogueira JB, Saeed SA, Askenazi DJ (2009). Rituximab therapy for severe refractory chronic Henoch-Schonlein purpura. J Pediatr.

[16] Bellan M, Pirisi M, Sainaghi PP (2016). Long-term remission of corticosteroid- and cyclophosphamide-resistant Henoch-Schonlein purpura with rituximab. Scand J Rheumatol.

[17] Eisenberg R, Looney RJ (2005). The therapeutic potential of anti-CD20 "what do B-cells do?". Clin Immunol.

[18] Hultin LE, Hausner MA, Hultin PM, Giorgi JV (1993). CD20 (pan-B cell) antigen is expressed at a low level on a subpopulation of human T lymphocytes. Cytometry.

[19] Levesque MC, St Clair EW (2008). B cell-directed therapies for autoimmune disease and correlates of disease response and relapse. J Allergy Clin Immunol.

[20] Tedder TF, Engel P (1994). CD20: a regulator of cell-cycle progression of B lymphocytes. Immunol Today.

[21] Quintanilla-Martinez L, Preffer F, Rubin D, Ferry JA, Harris NL (1994). CD20+ T-cell lymphoma. Neoplastic transformation of a normal T-cell subset. Am J Clin Pathol.

[22] Di Gaetano N, Cittera E, Nota R, Vecchi A, Grieco V, Scanziani E (2003). Complement activation determines the therapeutic activity of rituximab in vivo. J Immunol.

[23] Martin F, Chan AC (2006). B cell immunobiology in disease: evolving concepts from the clinic. Annu Rev Immunol.

[24] Cheifetz A, Mayer L (2005). Monoclonal antibodies, immunogenicity, and associated infusion reactions. Mt Sinai J Med.

[25] D'Arcy CA, Mannik M (2001). Serum sickness secondary to treatment with the murine-human chimeric antibody IDEC-C2B8 (rituximab). Arthritis Rheum.

[26] van der Kolk LE, Grillo-Lopez AJ, Baars JW, Hack CE, van Oers MH (2001). Complement activation plays a key role in the side-effects of rituximab treatment. Br J Haematol.

[27] Wang SY, Racila E, Taylor RP, Weiner GJ (2008). NK-cell activation and antibody-dependent cellular cytotoxicity induced by rituximab-coated target cells is inhibited by the C3b component of complement. Blood.

[28] Andersohn F, Konzen C, Garbe E (2007). Systematic review: agranulocytosis induced by nonchemotherapy drugs. Ann Intern Med.

[29] Arnold DM, Dentali F, Crowther MA, Meyer RM, Cook RJ, Sigouin C (2007). Systematic review: efficacy and safety of rituximab for adults with idiopathic thrombocytopenic purpura. Ann Intern Med.

[30] Nitta E, Izutsu K, Sato T, Ota Y, Takeuchi K, Kamijo A (2007). A high incidence of late-onset neutropenia following rituximab-containing chemotherapy as a primary treatment of CD20-positive B-cell lymphoma: a single-institution study. Ann Oncol.

[31] Todd DJ, Helfgott SM (2007). Serum sickness following treatment with rituximab. J Rheumatol.

[32] Kronbichler A, Windpessl M, Pieringer H, Jayne DRW (2017). Rituximab for immunologic renal disease: what the nephrologist needs to know. Autoimmun Rev.

[33] Lu TY, Ng KP, Cambridge G, Leandro MJ, Edwards JC, Ehrenstein M (2009). A retrospective seven-year analysis of the use of B cell depletion therapy in systemic lupus erythematosus at University College London Hospital: the first fifty patients. Arthritis Rheum.

[34] Vinod SS, Reed AB, Maxwell J, Cron RQ, Stoll ML (2018). Pediatric rheumatology infusion center: report on therapeutic protocols and infusions given over 4 years with focus on adverse events over 1 year. Pediatr Rheumatol Online J..

[35] Petty RE, Southwood TR, Manners P, Baum J, Glass DN, Goldenberg J (2004). International league of associations for rheumatology classification of juvenile idiopathic arthritis: second revision, Edmonton, 2001. J Rheumatol.

[36] Tambralli A, Beukelman T, Cron RQ, Stoll ML (2015). Safety and efficacy of rituximab in childhood-onset systemic lupus erythematosus and other rheumatic diseases. J Rheumatol.

[37] Taha R, El-Haddad H, Almuallim A, Alshaiki F, Obaid E, Almoallim H (2017). Systematic review of the role of rituximab in treatment of antineutrophil cytoplasmic autoantibody-associated vasculitis, hepatitis C virus-related cryoglobulinemic vasculitis, Henoch-Schonlein purpura, ankylosing spondylitis, and Raynaud's phenomenon. Open Access Rheumatol.

[38] Pillebout E, Rocha F, Fardet L, Rybojad M, Verine J, Glotz D (2011). Successful outcome using rituximab as the only immunomodulation in Henoch-Schonlein purpura: case report. Nephrol Dial Transplant.

[39] Kostik MM, Chikova IA, Solovyev AA, Fedotova EP, Kalashnikova OV, Nasyrov RA (2013). The initial effectiveness of rituximab for nephrotic syndrome in severe pediatric Henoch-Schonlein glomerulonephritis. Ann Pediatr Rheumatol.

[40] Tosounidou S, Sahni M, Carruthers D (2013). AB0775 Has rituximab (RTX) a role in treatment of chronic non-renal henoch-schonlein purpura (HSP)? 9 years follow up of a patient with severe gastro-intestinal disease. Ann Rheum Dis.

[41] El-Husseini A, Ahmed A, Sabucedo A, Fabulo E (2013). Refractory Henoch-Schonlein purpura: atypical aetiology and management. J Ren care.

[42] Ishiguro H, Hashimoto T, Akata M, Suzuki S, Azushima K, Kobayashi Y (2013). Rituximab treatment for adult purpura nephritis with nephrotic syndrome. Intern Med.

[43] Pindi Sala T, Michot J-M, Snanoudj R, Dollat M, Estève E, Marie B, et al. Successful outcome of a corticodependent Henoch-Schönlein purpura adult with rituximab. Case Rep Med. 2014;2014. 10.1155/2014/619218 PMC3988713 24799911

